# Carcinoembryonic Antigen: Beyond a Gastrointestinal Tumour Marker

**DOI:** 10.7759/cureus.103182

**Published:** 2026-02-07

**Authors:** Aye Aye Tun, Chiaw Yuan Tan, Kosasih Sumitro, Fakhruddin Salim, Kian Chai Lim, Alice Yong, Vui Heng Chong

**Affiliations:** 1 Internal Medicine, Raja Isteri Pengiran Anak Saleha Hospital, Bandar Seri Begawan, BRN; 2 Otolaryngology, Raja Isteri Pengiran Anak Saleha Hospital, Bandar Seri Begawan, BRN; 3 Radiology, Raja Isteri Pengiran Anak Saleha Hospital, Bandar Seri Begawan, BRN

**Keywords:** carcinoembryonic antigen, gastroenterology referral, genetic testing, medullary thyroid carcinoma, ret proto-oncogene

## Abstract

Carcinoembryonic antigen (CEA) is a widely used, non-specific tumour marker for gastrointestinal (GI) malignancies, particularly colorectal cancer (CRC). However, it can also be elevated in non-GI tumours and benign conditions, which are often overlooked.

A 60-year-old, asymptomatic man was referred for assessment of GI malignancy. During a health screening check, he was found to have a markedly elevated CEA, 121.8 ng/mL (reference range: <5.0 ng/mL). His family history included CRC, breast, and thyroid cancers. GI evaluation, which included a colonoscopy and gastroscopy, identified only a small sigmoid polyp and *Helicobacter pylori* gastritis. A pan-computed tomography (CT) scan showed a left thyroid nodule. He had fine-needle aspiration biopsies of the thyroid nodule on two occasions, and both were negative for malignancy. Serum calcitonin was markedly elevated. Following a discussion, the patient underwent total thyroidectomy, which confirmed multifocal medullary thyroid carcinoma (MTC). Postoperatively, the serum CEA declined and normalised after four months. Genetic testing revealed a germline RET mutation, establishing hereditary MTC. CEA elevation is commonly evaluated for GI malignancies.

This case report highlights that MTC is also associated with elevated CEA. Therefore, this should be assessed when GI evaluations are negative, to avoid delay in diagnosis.

## Introduction

Carcinoembryonic antigen (CEA) is a widely used, non-specific tumour marker for gastrointestinal (GI) malignancies, particularly colorectal cancer (CRC) [1,2]. CEA, a glycoprotein, is normally derived from fetal embryonic endodermal epithelium and was first detected in colon cancer cells by Freedman and Gold [3]. It usually disappears from the serum after birth, but a small quantity may remain in the colon tissue. It is slightly higher in men than in women. CEA, which is high in fetal life, is measured at normal levels in pregnant women because it cannot cross the placenta [1,4,5]. CEA is predominantly metabolised in the liver [1]. False positive elevation of CEA levels is associated with hepatic and biliary dysfunction, as it slows down the metabolic process of CEA breakdown [1]. A high first-pass hepatic metabolism results in significantly elevated levels corresponding to CEA-producing tumours or metastases outside the portal venous drainage territory [6]. Despite its established role in GI cancers, CEA lacks specificity and can be elevated in non-GI cancers, as well as benign conditions, such as chronic obstructive pulmonary disease, smoking, pancreatitis, and cirrhosis [2,3,7]. We report an interesting case to highlight the importance of considering non-GI malignancy in patients with elevated CEA.

## Case presentation

A 60-year-old, asymptomatic man was referred to the Gastroenterology Unit for evaluation of GI malignancy. During a health screening, he was found to have a markedly elevated serum CEA, 121.8 ng/mL (reference range: <5 ng/mL). He also had a strong family history of cancer, which included CRC, breast, and thyroid cancers among siblings. He denied smoking. Physical examination revealed him to be of thin build, without cutaneous abnormalities, and system examination was otherwise normal.

Laboratory investigations, which included stool occult blood, complete blood count, and renal, liver, and thyroid profiles, were negative apart from the CEA. Other tumour markers - carbohydrate antigen 19-9 (CA 19-9), alpha-fetoprotein (AFP), and prostate-specific antigen (PSA) - were normal. Point-of-care ultrasound of the abdomen, done in the clinic, was normal and did not show any obvious abnormalities of the solid and hollow organs.

**Figure 1:**
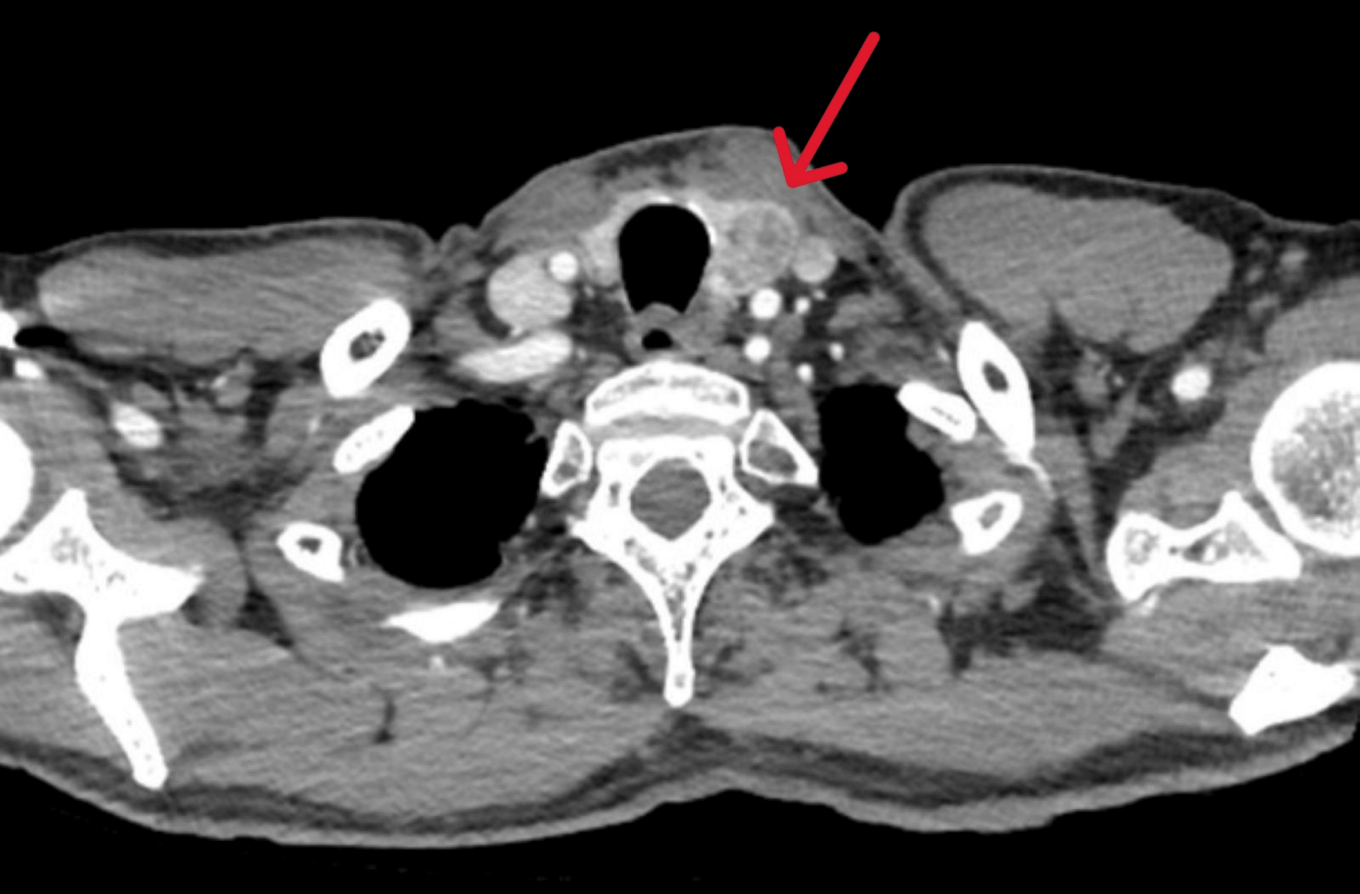
Axial CT image showing a left thyroid nodule (arrow). CT, Computed tomography

He proceeded with GI endoscopies, which showed only a small sigmoid polyp, and upper GI endoscopy revealed *Helicobacter pylori* gastritis, which was successfully treated. A pan-computed tomography (CT) scan was done, and this did not show any GI or pulmonary pathology but incidentally detected a left thyroid nodule (Figure 1) and a right thyroid cyst.

**Figure 2:**
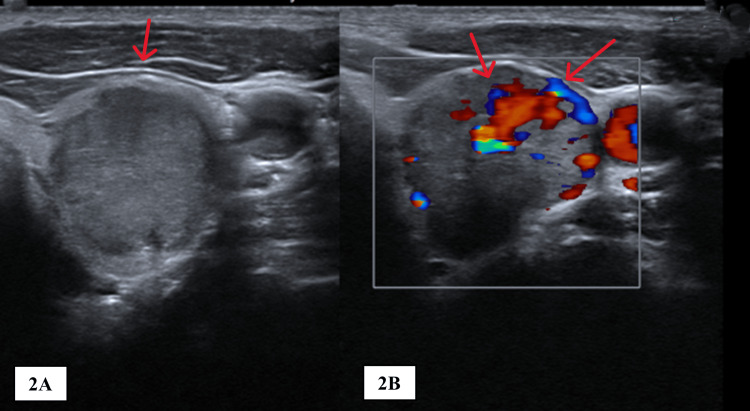
Ultrasound scan of the thyroid showing a nodule (A, arrow), a nodule with increased vascularity (B, arrows).

An ultrasound to assess the thyroid nodule showed a round nodule with increased vascularity (Figure 2). Because of the non-yielding investigations, the patient proceeded with a positron emission tomography-computed tomography (PET-CT), which showed hypermetabolic activity in the left thyroid lobe, corresponding to the thyroid nodule.

In the meantime, the patient was also referred to and seen by the Otorhinolaryngologist. In view of the findings, a fine-needle aspiration cytology was done, which was negative for malignancy. Serum calcitonin was markedly elevated, 1,924 pg/mL (reference range: <14.3 pg/mL). The serum CEA continued to be elevated, and after discussion, the patient had a repeat fine-needle aspiration, which was again non-diagnostic.

As the serum CEA level remained persistently elevated, along with high calcitonin, thyroid malignancy was suspected - specifically medullary thyroid carcinoma (MTC) - and a multidisciplinary team discussion on thyroidectomy was conducted. This was later discussed with the patient, who agreed to proceed with a total thyroidectomy with central neck dissection. The patient subsequently underwent total thyroidectomy with selective neck dissection, which was completed without immediate intraoperative complications.

Histopathological examination confirmed multifocal MTC, with the largest tumour focus measuring 25 mm. Metastatic involvement was identified in the central compartment lymph nodes, with no evidence of lateral neck metastasis. The final pathological staging was pT2(m) pN1a M0.

**Figure 3:**
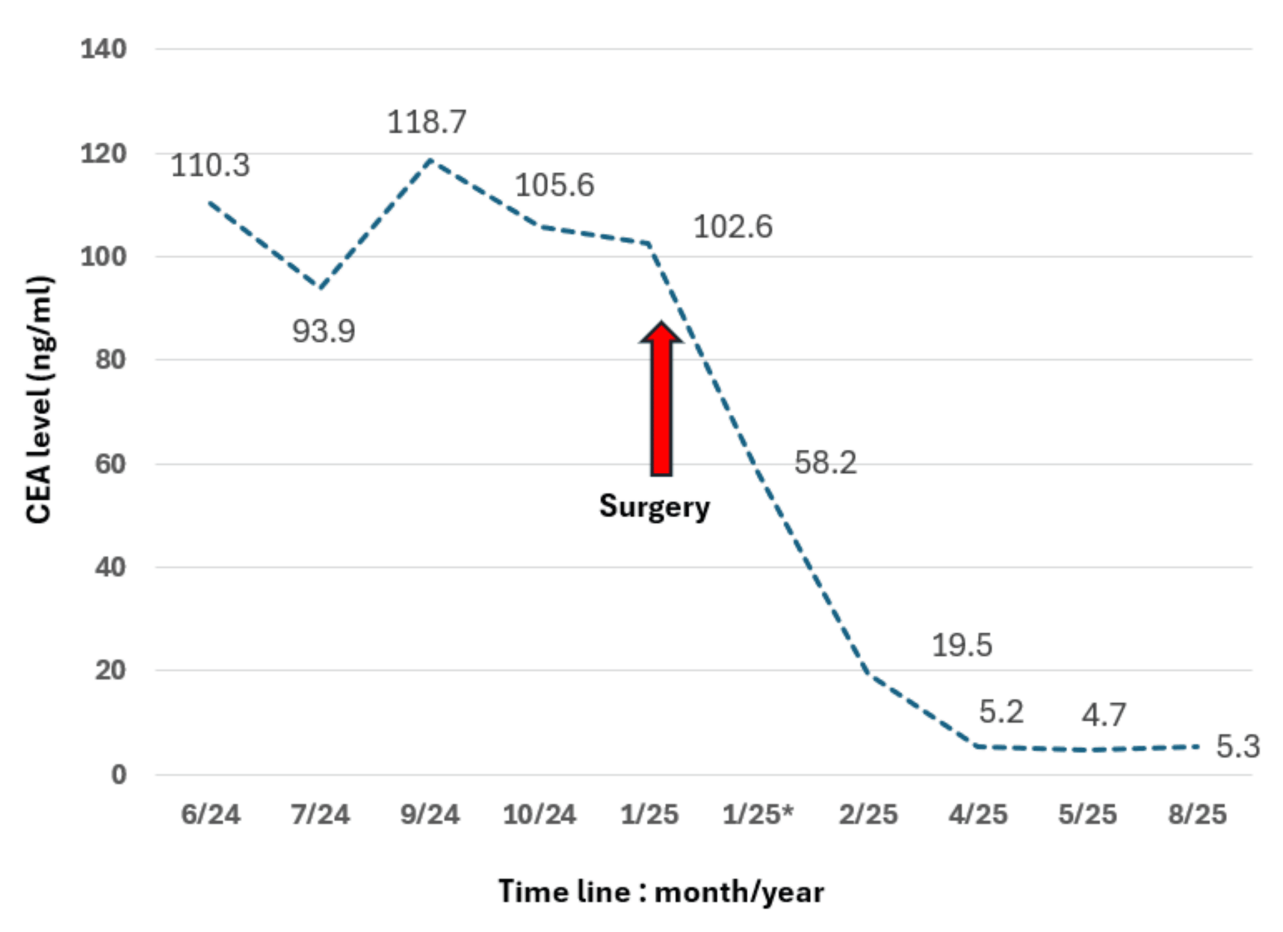
Timeline showing trend of serum CEA levels. 1/25* - nine days after surgery. CEA, Carcinoembryonic antigen

On follow-up, the patient remained well, and monitoring of the serum CEA showed a progressive decline to a normal level (Figure 3). The CEA levels normalised (5/2025 - 4.7; normal range <5.0 ng/mL) within four months post-surgery. On follow-up, the level increased slightly, but the patient remained clinically well, and there was no ultrasound evidence of recurrence. He continued to be monitored.

## Discussion

CEA is a serum tumour biomarker that is commonly associated with GI malignancies, especially CRC [1,2]. However, it can also be elevated in many other conditions - malignant and benign (Table 1) - including benign tumours, usually in organs where cancers are associated with elevated CEA levels [1-3,6,7]. Hence, serum CEA elevation is not a definitive marker of any cancer site of origin [3]. In malignant conditions, elevated CEA can be seen in any tumour of epithelial origin, especially in the GI tract [2]. Elevated CEA is also well documented in MTC [8-10]. Normal serum CEA values are generally referenced as <5 ng/mL in adult non-smokers and can be higher in smokers, but typically <10 ng/mL [1]. Serum CEA levels are <2.5 ng/mL in 85% of adults and <5 ng/mL in 95% [1]. Generally, serum levels of more than 10.0 ng/mL, or trending upward, are commonly associated with malignant conditions [2], and when the level is much higher, as in our patient, malignancies are often present and need to be thoroughly investigated.

**Table 1:** Conditions associated with elevated CEA. Source: [1,2] CEA, Carcinoembryonic antigen

Category	Malignant	Benign
Gastrointestinal	Colorectal, oesophageal, and gastric cancer	Peptic ulcer disease, inflammatory bowel disease, diverticulitis
Pancreato-hepatobiliary	Pancreatic cancer, cholangiocarcinoma	Pancreatitis, cirrhosis, chronic liver disease
Pulmonary	Non-small cell lung cancer	Smoking
Dermatologic	Melanoma	Dermatitis
Endocrine	Medullary thyroid carcinoma, breast cancer	Hypothyroidism, fibrocystic breast disease
Genitourinary	Prostate cancer, ovarian cancer, and mucinous adenocarcinoma of the cervix	-
Others	Choriocarcinoma, osteosarcoma, retinoblastoma, lymphoma	Infections

In practice, patients with elevated CEA are often referred for a GI malignancy workup [2] and hence are generally referred to the gastroenterology service. This consists of a thorough history and physical examination, followed by evaluation of the GI tract, commonly including colonoscopy and gastroscopy. After negative endoscopies, clinicians either proceed with further evaluation - typically with whole-body imaging such as a CT scan or PET scan - or elect to monitor the CEA trends, especially in the absence of signs, symptoms, or a history of cancer [2]. However, monitoring can lead to a delay in diagnosis. Our case highlights the importance of proceeding with evaluating other causes, which can be myriad. In our case, the pan-CT scan excluded hepatobiliary, pancreatic, and pulmonary causes. The imaging incidentally detected a thyroid nodule. It was only after negative evaluations of GI and pulmonary causes that the thyroid was investigated. Elevated calcitonin and a family history of thyroid cancer suggested a causal association. Interestingly, it later came to light that the thyroid cancer that the patient’s sister had was also MTC.

MTC is a rare thyroid malignancy, accounting for 3%-5% of cases. Approximately 75%-80% of cases are sporadic, while 20%-25% are hereditary, most commonly associated with multiple endocrine neoplasia type 2A (MEN2A) or multiple endocrine neoplasia type 2B (MEN2B) [8]. MTC arises from parafollicular C-cells, which secrete calcitonin, but CEA may also be elevated and, sometimes, is the initial abnormal finding that prompts referral [8-10]. MTC has distinct clinical and genetic characteristics. Unlike papillary and follicular thyroid cancers, which arise from follicular cells, MTC originates from parafollicular C-cells, leading to the secretion of both calcitonin and CEA. Elevated calcitonin is the most sensitive biomarker, but CEA may also be markedly raised, occasionally prompting referral to gastroenterology. Early recognition is crucial, as hereditary MTC is often associated with multiple endocrine neoplasia type 2 (MEN2) and carries important implications for family screening [1].

Early diagnosis is critical, as hereditary MTC has implications for genetic counselling, family screening, and timely prophylactic surgery in at-risk carriers [11,12]. This case highlights the need for a multidisciplinary approach to unexplained tumour marker abnormalities.

## Conclusions

Although commonly associated with GI malignancies, elevated CEA can be due to other pathologies - benign and malignant - including MTC. Persistent unexplained CEA elevation, particularly in patients with a relevant family history, should prompt evaluation for other malignancies, such as MTC. A broad diagnostic approach ensures timely treatment and enables family risk stratification.

## References

[1] Dilek ON, Arslan Kahraman Dİ, Kahraman G (2024). Carcinoembryonic antigen in the diagnosis, treatment, and follow-up of focal liver lesions. World J Gastrointest Surg.

[2] Hall C, Clarke L, Pal A, Buchwald P, Eglinton T, Wakeman C, Frizelle F (2019). A review of the role of carcinoembryonic antigen in clinical practice. Ann Coloproctol.

[3] Perkins GL, Slater ED, Sanders GK, Prichard JG (2003). Serum tumor markers. Am Fam Physician.

[4] Goslin R, O'Brien MJ, Steele G, Mayer R, Wilson R, Corson JM, Zamcheck N (1981). Correlation of plasma CEA and CEA tissue staining in poorly differentiated colorectal cancer. Am J Med.

[5] Fukuda I, Yamakado M, Kiyose H (1998). Influence of smoking on serum carcinoembryonic antigen levels in subjects who underwent multiphasic health testing and services. J Med Syst.

[6] Vijaya LK, Muhammad Z, Shiva KR (2025). Carcinoembryogenic antigen. StatPearls [Internet].

[7] Locker GY, Hamilton S, Harris J (2006). ASCO 2006 update of recommendations for the use of tumor markers in gastrointestinal cancer. J Clin Oncol.

[8] Wells SA Jr, Asa SL, Dralle H (2015). Revised American Thyroid Association guidelines for the management of medullary thyroid carcinoma. Thyroid.

[9] Kebebew E, Ituarte PH, Siperstein AE (2000). Medullary thyroid carcinoma: clinical characteristics, treatment, prognostic factors, and a comparison of staging systems. Cancer.

[10] Kloos RT, Eng C, Evans DB (2009). Medullary thyroid cancer: management guidelines of the American Thyroid Association. Thyroid.

[11] Elisei R, Alevizaki M, Conte-Devolx B, Frank-Raue K, Leite V, Williams GR (2013). 2012 European Thyroid Association guidelines for genetic testing and its clinical consequences in medullary thyroid cancer. Eur Thyroid J.

[12] Machens A, Dralle H (2009). Prophylactic thyroidectomy in RET carriers at risk for hereditary medullary thyroid cancer. Thyroid.

